# Effect of Local Administration of Meglumine Antimoniate and Polyhexamethylene Biguanide Alone or in Combination with a Toll-like Receptor 4 Agonist for the Treatment of Papular Dermatitis due to *Leishmania infantum* in Dogs

**DOI:** 10.3390/pathogens12060821

**Published:** 2023-06-10

**Authors:** Icíar Martínez-Flórez, Maria Jose Guerrero, Annabel Dalmau, Maria Cabré, Maria Magdalena Alcover, Diana Berenguer, Liam Good, Roser Fisa, Cristina Riera, Laura Ordeix, Laia Solano-Gallego

**Affiliations:** 1 Departament de Medicina i Cirurgia Animals, Universitat Autònoma de Barcelona, 08193 Cerdanyola del Vallès, Spain; imflorez.vet@gmail.com (I.M.-F.); mariacabregil@gmail.com (M.C.); laura.ordeix@uab.cat (L.O.); 2 Hospital Veterinario Alhaurín el Grande, 29120 Alhaurín el Grande, Spain; marjoxgr@gmail.com; 3 AniCura Mediterrani Hospital Veterinari, 43204 Reus, Spain; annabeldalmaulopez@gmail.com; 4 Fundació Hospital Clínic Veterinari, Universitat Autònoma de Barcelona, 08193 Cerdanyola del Vallès, Spain; 5 Departament de Biologia, Sanitat i Medi Ambient, Secció de Parasitologia, Facultat de Farmàcia i Ciències de l’Alimentació, Universitat de Barcelona, 08028 Barcelona, Spain; mmagdalenaalcoveramengual@ub.edu (M.M.A.); berenguer.diana@gmail.com (D.B.); rfisa@ub.edu (R.F.); mcriera@ub.edu (C.R.); 6 Department of Pathobiology and Population Sciences, The Royal Veterinary College, University of London, London NW1 0TU, UK; lgood@rvc.ac.uk

**Keywords:** canine, cutaneous lesions, immunotherapy, leishmaniosis, local treatment, toll-like agonist

## Abstract

Papular dermatitis is a cutaneous manifestation of canine *Leishmania infantum* infection associated with mild disease. Although it is a typical presentation, nowadays, there is still no established treatment. This study evaluated the safety and clinical efficacy of local meglumine antimoniate, locally administered polyhexamethylene biguanide (PHMB) alone or PHMB in combination with a Toll-like receptor 4 agonist (TLR4a) for the treatment of papular dermatitis due to *L. infantum* and assessed parasitological and immunological markers in this disease. Twenty-eight dogs with papular dermatitis were divided randomly into four different groups; three of them were considered treatment groups: PHMB (n = 5), PHMB + TLR4a (n = 4), and meglumine antimoniate (n = 10)), and the remaining were considered the placebo group (n = 9), which was further subdivided into two sub-groups: diluent (n = 5) and TLR4a (n = 4). Dogs were treated locally every 12 h for four weeks. Compared to placebo, local administration of PHMB (alone or with TLR4a) showed a higher tendency towards resolution of papular dermatitis due to *L. infantum* infection at day 15 (χ^2^ = 5.78; df = 2, *p* = 0.06) and day 30 (χ^2^ = 4.; df = 2, *p* = 0.12), while local meglumine antimoniate administration demonstrated the fastest clinical resolution after 15 (χ^2^ = 12.58; df = 2, *p* = 0.002) and 30 days post-treatment (χ^2^ = 9.47; df = 2, *p* = 0.009). Meglumine antimoniate showed a higher tendency towards resolution at day 30 when compared with PHMB (alone or with TLR4a) (χ^2^ = 4.74; df = 2, *p* = 0.09). In conclusion, the local administration of meglumine antimoniate appears to be safe and clinically efficient for the treatment of canine papular dermatitis due to *L. infantum* infection.

## 1. Introduction

Canine leishmaniosis (CanL) is a vector-borne zoonotic disease with a worldwide distribution caused by the protozoan parasite *Leishmania infantum*. It is considered an endemic disease in the Mediterranean basin, Portugal, Latin America, and southern Asia [1]. Female phlebotomine sand flies are the biological vector, and dogs are considered the main reservoir [2].

Canine leishmaniosis is manifested by a broad spectrum of clinical signs and a wide range of disease severity that is classified into four stages: mild (stage I), moderate (stage II), severe (stage III), or very severe disease (stage IV) based on clinical signs, clinicopathological abnormalities, and measurement of anti-leishmanial antibodies [1]. Cutaneous manifestations are the most common clinical signs, and among them, papular dermatitis is considered a typical presentation in endemic areas and is associated with Leishvet stage I-mild leishmaniosis [1,3]. This cutaneous form is associated with an effective Th1 predominant parasite-specific cellular immunity and low humoral immune response, and thus, it is characterized by the absence of laboratory abnormalities and lack of systemic clinical signs. Self-healing of the skin lesions may occur at some point; however, it usually takes three to six months [4].

Clinical signs and outcomes of CanL depend on the interactions between the parasite and the host’s innate and adaptive immune responses [5,6,7]. The adaptive response characterized by T-helper 1 (Th1) activation produces cytokines such as interferon-gamma (IFN-γ), interleukin-2 (IL-2), and tumor necrosis factor-alpha (TNF-α), which help to control the infection. In contrast, the immune response mediated by Th2 induces the production of anti-inflammatory cytokines such as interleukin-4 (IL-4), interleukin-10 (IL-10), and transforming growth factor-beta (TGF-β) and is associated with disease progression [7,8]. Interleukin-17a (IL-17a) produced by several cells such as Th17, natural killer cells (NK), and macrophages, among others [9], plays a role in blocking parasite growth by the activation of inducible nitric oxide synthase (iNOS), among others [10,11]. Therefore, IL-17a is associated with resistance against *L. infantum* infection or disease in humans [10] and dogs [12], acting synergistically with IFN-γ.

Regarding the Th1 response, disease control requires a balanced interaction between a proinflammatory immune response mediated by T helper 1 type (Th1) CD4+ T cells, with the aim of controlling parasite replication, and a regulatory immune response mediated by T regulatory 1 cells, which are necessary to avoid a self-damaging overactivation of the immune system [13]. B cells also play a key role in the progression of CanL. They induce a humoral immune response and act as antigen-presenting cells modulating the activation of CD4+ T cells, which in turn activate B cells to produce antibodies [13]. As the disease progresses, the production of specific and non-specific IgG antibodies increases, reflected as hypergammaglobulinemia, and binds to *Leishmania* antigen creating immune complexes, which may contribute to disease progression [14,15,16].

One of the factors that regulate the activation of Th1 or Th2 immunity is pattern recognition receptors (PRRs) such as Toll-like receptors (TLRs), which recognize molecules found in the promastigote that act as pathogen-associated molecular patterns (PAMPs) [17]. TLRs are located on the membrane or intracellular compartments of different types of cells, including T and B lymphocytes, macrophages, dendritic cells (DCs), and NK [18]. After binding their ligand, TLRs trigger the activation of the immune activity through diverse events such as the induction of inflammatory cytokines such as TNF-α and IFN-γ [19,20,21,22].

The innate immune response has an essential role in avoiding *Leishmania* survival. Cells such as neutrophils, macrophages, and DCs can kill the parasite via phagocytosis or facilitate their elimination by producing cytokines [7]. Neutrophils are activated following *L. infantum* inoculation and initiate several defense mechanisms, such as the production of reactive oxygen species (ROS) [23]. The nitroblue tetrazolium (NBT) reduction test is used to detect activated neutrophils in peripheral blood. NBT (soluble and colorless) is transformed into formazan (insoluble and blue-grey) in activated neutrophils, shown to be directly related to ROS production [24,25].

Treatment regarding Leishvet stage I has been scientifically neglected. Short-term treatment with one or two conventional anti-*Leishmania* drugs (meglumine antimoniate, miltefosine and/or allopurinol) in dogs classified as Leishvet stage I has been described, as well as immune-potentiating treatments (domperidone or nucleotides plus- active hexose correlated compound (AHCC)) alone or in combination with the previously mentioned drugs [1,26]. Alternatively, monitoring without treatment can also be considered in some cases. There is limited evidence for treatment outcomes for dogs in this stage, and therefore, the clinical efficacy of these treatment options remains unknown [27].

Similarly, there is no universally applicable treatment for human cutaneous leishmaniasis (CL) [28]. Local treatment for CL in humans has been recommended as an alternative treatment to systemic therapy by the World Health Organization and the Pan American Health Organization in patients with at least four lesions smaller than 4 cm in diameter, especially if the face and joints are not affected [29]. Local treatments are described as easy to use and with a lower risk, toxicity, and cost compared to traditional therapies, and they include intralesional antimonial injections, cryotherapy (with liquid nitrogen), thermotherapy (use of localized current field radiofrequency heat), and topical formulations such as paromomycin ointment [28,30,31,32]. However, local treatment has not been investigated in stage I-papular dermatitis due to canine *L. infantum* infection.

Polyhexamethylene biguanide (PHMB) is a synthetic cationic polymer with antimicrobial activity commonly used as a first-line treatment for locally infected wounds [33]. Furthermore, PHMB has been reported to have antileishmanial activity by inducing disruption of the parasite´s membrane integrity and causing chromosomal damage [34]. On the other hand, TLR4 activation induces a protective immune response against *Leishmania*, and will also regulate iNOS leading to the death of the parasite [22]. PHMB alone or in combination with a TLR4 agonist (TLR4a) has been reported to induce lower percentages of *Leishmania* infection in canine DH-82 cells in vitro [22].

The main aim of this randomized, controlled, double-blinded study was to evaluate the safety and clinical efficacy of locally administered PHMB alone or in combination with TLR4a and the local administration of meglumine antimoniate in the treatment of papular dermatitis due to *L. infantum* infection in dogs. The secondary objectives were to assess parasite-specific humoral and cellular immunity as well as parasitemia and to evaluate neutrophil function based on NBT at the time of diagnosis and during the follow-up period.

## 2. Materials and Methods

### 2.1. Dogs and Study Design

This study was designed as a multicentric randomized double-blinded controlled study with a follow-up of one year and involving five veterinary centers from Spain: Hospital Veterinario Alhaurín El Grande (Málaga), Hospital Mediterrani Veterinaris (Reus, Tarragona), Clínica veterinaria Paws Patas (Mula, Murcia), Hospital Veterinari Canis (Palma, Mallorca), and Fundació Hospital Clínic Veterinari (UAB, Bellaterra, Barcelona).

Dogs visited at the mentioned centers from 2019 to 2022 were considered for inclusion in this study. Inclusion criteria were a diagnosis of mild leishmaniosis (LeishVet stage I) characterized by the absence of laboratory abnormalities, negative or low antibody levels, and papular dermatitis as the only clinical sign at the time of diagnosis [1]. Withdrawal criteria were the development of systemic disease based on clinical signs and clinicopathological abnormalities, treatment with conventional anti-*Leishmania* drugs (meglumine antimoniate, miltefosine, or allopurinol), or highly increased antibody levels. A signed informed consent was obtained from all dog owners. Ethical approval was obtained by “Comissió d’Ètica en l’Experimentació Animal i Humana de la Universitat Autònoma de Barcelona” (CEAAH 4526, November 2018) and by “Generalitat de Catalunya” (FUE-2018-00944112 i ID KSHYD6LVR, April 2019).

A complete physical examination, complete blood count (CBC), and biochemistry panel, including at least creatinine, urea, total proteins (TP), and alanine transaminase (ALT), were performed in all dogs to assess the clinical status. In some dogs, serum electrophoresis and urinalysis with urinary protein/creatinine ratio were also performed. Cutaneous lesions were photographed, borders around each lesion were measured to document size, and a cytology examination was performed from the lesions to diagnose *L. infantum* infection.

### 2.2. Treatment and Follow-Up

Five different products were manufactured as follows: PHMB alone (3 mg/mL PHMB in 30% ethanol), a combination of PHMB and TLR4a (1.5 µg/mL TLR4 agonist + 3 mg/mL PHMB in 30% ethanol), TLR4a alone (1.5 µg/mL TLR4 agonist in 30% ethanol), meglumine antimoniate (30% meglumine antimoniate + 70% pluronic F-127), and diluent (30% ethanol in nuclease-free water).

PHMB, PHMB + TLR4a, TLR4a, and diluent (30% ethanol in nuclease-free water) were formulated as liquid spray formulations. It was strongly advised to gently invert 20 times the sprayer vial before every application and to keep it upright when spraying.

Meglumine antimoniate was a lotion spray formulation and was stored in the refrigerator. It was recommended to remove the remaining product over the patient’s skin from the last application before a new administration [35].

**Figure 1:**
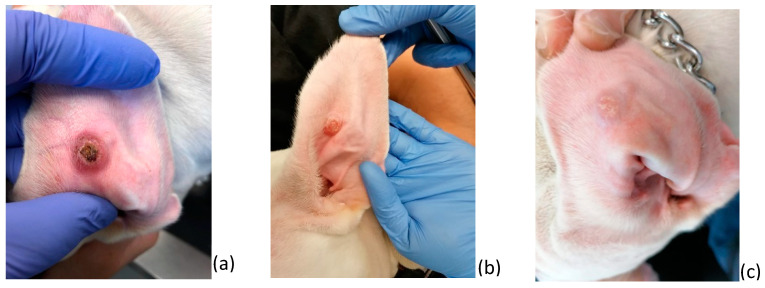
Clinical efficacy of local PHMB on papular dermatitis due to *L. infantum* at day 0 (**a**), day 15 (**b**) showing partial remission, and day 30 (**c**) full remission. Courtesy of Isaac Carrasco.

**Figure 2:**
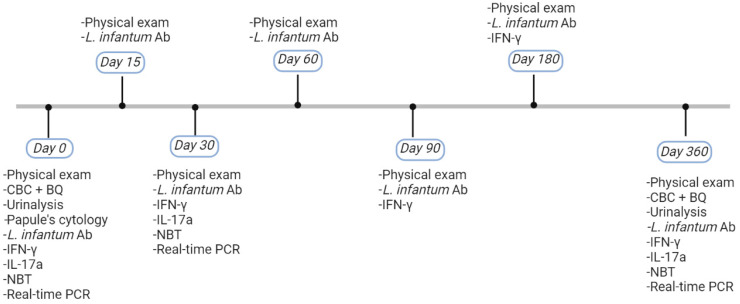
Timeline of the procedures performed on each dog at the time of diagnosis and during follow-up. Created with BioRender.com. CBC = complete blood count, BQ = biochemistry panel, Ab = antibody, IFN-γ = Interferon-gamma, IL-17a = Interleukin-17a, LSA = soluble *L. infantum* antigen, NBT = Nitroblue tetrazolium.

Dogs were randomly divided into four groups: three of them were considered treatment groups, PHMB (n = 5), PHMB + TLR4a (n = 4), and meglumine antimoniate (n = 10), and the remaining was considered the placebo group (n = 9), which was further subdivided into two sub-groups (diluent (n = 5) and TLR4a (n = 4)). TLR4a showed no statistical differences when compared to a diluent, and therefore, both groups were considered as placebo. Each group was treated locally over the papules every 12 h for four weeks, and a physical exam and lesion follow-up were performed on days 15, 30, 60, 90, 180, and 365. Clinical efficacy was defined as partial or total resolution of the lesions at days 15 and 30, respectively (Figure 1). All the procedures performed on each dog at the time of diagnosis and during follow-up are described at Figure 2.

Persistent papules after sixty days from the beginning of the study or the appearance of new papules during the treatment were considered as a lack of response to treatment and relapse, respectively, and a rescue therapy (topical administration of 30% meglumine antimoniate + 70% pluronic F-127) was administered.

### 2.3. Safety Assessment

Drug safety assessment was performed based on a complete physical exam on days 15, 30, and 60 by veterinarians. Local side effects of the topical treatments at the application site, such as erythema and swelling, were recorded and graded (0–4) (Supplementary Material Table S1). Local pruritus and pain were also recorded. Systemic signs such as lymphadenomegaly or increased temperature, among others, were also registered. This information was recorded in the data collection form of both the veterinarian and the owner or caregiver of the dog (Supplementary Material Table S1). Furthermore, photographs of the application site were also reviewed by the first author to check for local reactions on days 15, 30, and 60.

### 2.4. ELISA for specific L. infantum Antibody Detection

*Leishmania infantum* antibody levels were measured by quantitative serology using an in-house Enzyme-Linked ImmunoSorbent Assay (ELISA) at the time of diagnosis and repeated on days 15, 30, 60, 90, 180, and 365 [5].

The in-house ELISA was performed on the sera of all dogs as previously described [36]. Briefly, the samples were diluted to 1:800 in phosphate buffer solution (PBS) with Tween 20 and 1% dry milk and incubated at 37° for 1 h. Then, the plates were washed three times with PBS-Tween and once with PBS and incubated with Protein A conjugated to horseradish peroxidase (Peroxidase Conjugate Protein A; Merck KGaA, Darmstadt, Germany) at 1:30,000 dilution for 1 h at 37 °C. After incubation, the plates were washed again, as described before. Then, o-phenylenediamine and substrate buffer (SIGMAFAST OPD; Merck KGaA, Darmstadt, Germany) was added to the plates, and the reaction was finally stopped with 5 M H2SO4. Absorbance values were read at 492 nm in a spectrophotometer (MB-580 HEALES; Shenzhen Huisong Technology Development Co., Ltd., Shenzhen, China), and the results were quantified as ELISA units (EU) related to a positive canine serum used as a calibrator and set at 100 EU.

The cut-off of the serum in-house ELISA was already determined to be 35 EU using the ELISA results of 80 dogs from a non-endemic area, as previously described [37]. Cut-off was established by the standard deviation (SD) method, consisting of multiplying the SD of the results by four and adding up the mean of the results obtained by the ELISA (mean + 4 SD). Serum was classified as high positive when the result was ≥300 EU, medium positive when the result was ≥150 EU and <300 EU, low positive when the result was ≥35 EU and <150 EU, and negative when the result was <35 EU [37]. All samples from each animal were analyzed on the same plate.

### 2.5. Cytokine Release Whole Blood Assay and Determination of Canine IFN-γ and IL-17a

IFN-γ concentration was measured at admission in all dogs and repeated at days 30, 90, 180, and 365, while IL-17a was performed at days 0 and 30.

A heparinized whole blood cytokine release assay was performed as described elsewhere [5]. Briefly, whole blood was separately incubated with three different conditions: (i) medium alone (unstimulated); (ii) medium with soluble *L. infantum* antigen (LSA) at a concentration of 10 µg/mL; and (iii) medium with the mitogen concanavalin A (ConA, 100 mg, Medicago^®^, Uppsala, Sweden) at a concentration of 10 µg/mL. Blood cultures were collected after five days at 37 °C in 5% of CO_2_ and were centrifugated at 300 g for 10 min. Supernatants were collected and stored at −80 °C until tested. IFN-γ and IL-17a were determined in all samples by a commercial sandwich ELISA (DuoSet^®^ ELISA by Development System R&DTM, Abingdon, UK) [5,6]. The standard curve for IFN-γ and IL-17a was calculated using a computer-generated four-parameter logistic curve fit with the program MyAssays (http://www.myassays.com/) [5].

Dogs were classified as IFN-γ producers when *L. infantum*-specific IFN-γ concentration was ≥110 pg/mL after subtracting the medium alone [38]. In the case of IL-17a, dogs were classified as producers when *L. infantum*-specific IL-17a concentration was ≥62.5 pg/mL after subtracting the medium alone [5].

### 2.6. Nitroblue Tetrazolium Reduction Test

Hematocrit capillary microtubes were filled with blood samples collected in EDTA tubes from all dogs and centrifugated at 2910 g for 5 min. Then, the buffy coat from each microtube was placed in an Eppendorf tube with the same volume of 0.1% NBT solution (1:1) (N6876, Sigma–Aldrich Co., St. Louis, MO, USA). The Eppendorf tube was mildly agitated and incubated for 15 min at 37 °C and then for another 15 min at room temperature. Two blood smears from each Eppendorf tube were obtained by placing 3 µL of NBT-stained samples on each slide. The slides were stained with Diff-Quick, and the NBT reduction rate was assessed by light microscopy after counting 300 neutrophils. The percentage was calculated as the number of activated neutrophils, defined by those containing blue-black formazan deposits, divided by the total number of neutrophils counted, multiplying the result by 100.

### 2.7. Blood DNA Extraction and Leishmania Real-Time PCR

Real-time PCR was carried out from blood samples at day 0, day 30, and 6 or 12 months.

DNA was isolated from blood samples collected in EDTA tubes using MagMax CORE Nucleic Acid Purification Kit (Thermo Fisher Scientific Inc., Waltham, MA, USA) using an automated system (KingFisher Flex Purification System, Thermo Fisher Scientific Inc., Waltham, MA, USA) following the manufacturer’s instructions for a simple workflow with whole blood samples. Briefly, 10 µL of proteinase K solution and 20 µL of magnetic beads were added to 100 µL of each sample. DNA was extracted by the robot after adding 700 µL of a mix of the binding and lysis solution in all the samples [36].

*Leishmania* real-time PCR was performed as previously described [39]. Each DNA sequence amplification for PCR was performed in triplicate. The mix reaction was prepared with 1x iTaq supermix with Rox (Bio-Rad), 0.2 µmoL of direct primer (5′-CTT TTC TGG TCC TCC GGG TAGG-3′), 0.2 µmoL of reverse primer (5′-CCA CCC GGC CCT ATT TTA CAC CAA -3′), 20 nmol of the labeled TaqMan probe (FAM- TTT TCG CAG AAC GCC CCT ACC CGC TAMRA), 0.5 µL of H20, and 2.5 µL of sample DNA. Positive and negative controls were also included in each plate. A 10-fold dilution series of standard DNA from *L. infantum* promastigotes (CATB101) was used as a calibrator (serial dilution from 10^5^ parasites/mL to 10^−3^ parasites/mL), allowing for the plotting of a standard curve. Cycling was performed using the QuantStudio™ 7 Pro (Thermo Fisher Scientific, Foster City, CA, USA) at 95 °C/55 °C for 45 cycles. The PCR was considered positive for *Leishmania* when the quantification cycle (Cq) was <40, and the amplification was detected in all the replicates.

### 2.8. Statistical Analysis

The statistical analysis was performed using GraphPad Prism 8.0.1 for Windows software (GraphPad Software, San Diego, CA, USA). First, different normality tests were performed to know if the different variables (age, ELISA results, real-time PCR, LSA IFN-γ, ConA IFN-γ, LSA IL-17a, ConA IL-17a, and NBT rate) had a normal distribution at the time of diagnosis and follow-up. For each variable, the following tests were performed: Anderson–Darling, D’Agostino and Pearson, Shapiro–Wilk, and Kolmogorov–Smirnov. A *p*-value < 0.05 was considered statistically significant, meaning all variables did not follow a normal distribution except for the NBT rate. Therefore, non-parametric tests were used unless the comparison was between NBT rate groups, in which case, a parametric test was performed (paired t-test). A non-parametric Wilcoxon matched-pairs signed rank test was used for quantitative variables to compare two groups if the samples were paired. If samples were unpaired, a Mann–Whitney U-test was used. A Friedman test was used to compare more than two groups, and afterward, Dunn’s multiple comparisons test was performed. The chi-square test or Fisher´s exact test was used to determine whether there was a significant association between two categorical variables. The Spearman Correlation Coefficient was used to evaluate differences in cytokine production of the dogs studied. Differences were considered significant, with a 5% significance level (*p*-value < 0.05).

## 3. Results

### 3.1. Dog Characteristics at the Time of Diagnosis

**Figure 3:**
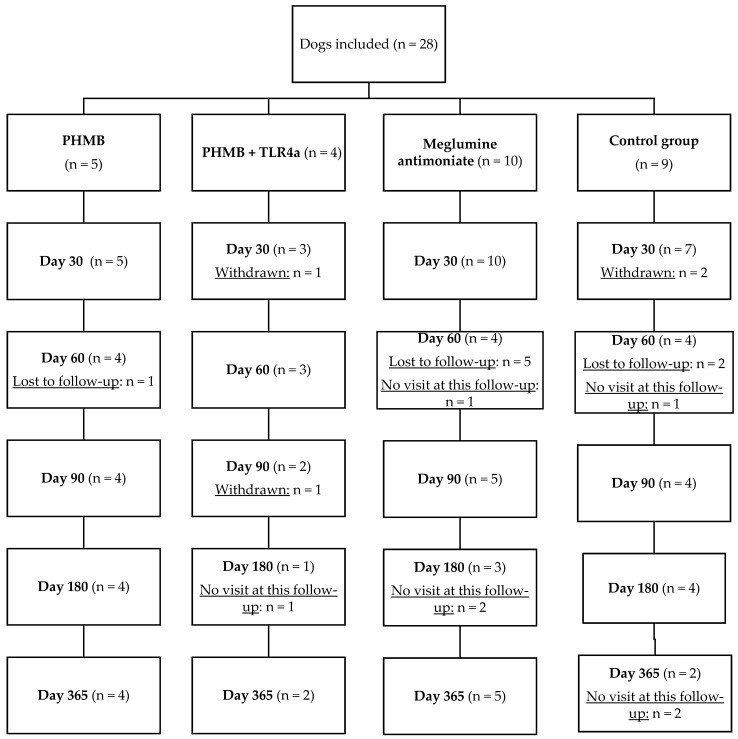
Flowchart displaying the number of dogs included at the time of diagnosis, treatment groups, as well as withdrawn dogs and lost to follow-up during the follow-up period. PHMB = polyhexamethylene biguanide, TLR4a = Toll-like receptor 4 agonist, Control group (n = 9): diluent (n = 5) + TLR4a (n = 4). Lost to follow-up = owners did not want to participate in the study anymore. No visit at this follow-up = owners did not show up at the Veterinary clinic that day. Withdrawn = dog was withdrawn of the study due to the development of systemic disease based on clinical signs and clinicopathological abnormalities, treatment with conventional anti-*Leishmania* drugs (meglumine antimoniate, miltefosine, or allopurinol), or highly increased antibody levels.

Twenty-eight dogs were included in this study (Figure 3) and were randomly divided into the aforementioned groups as follows: PHMB (n = 5), PHMB + TLR4a (n = 4), TLR4a-placebo (n = 4), meglumine antimoniate (n = 10), and diluent-placebo (n = 5). Unfortunately, complete blood samples and data were only obtained from 24, including 9 females and 15 males (37.5% and 62.5%, respectively).

Fifty-four percent of dogs were crossbred (n = 13); other represented breeds were Belgian Malinois (n = 4), German shepherd (n = 2), American bully (n = 1), Border collie (n = 1), Chihuahua (n = 1), Labrador retriever (n = 1), and Rottweiler (n = 1). Their ages ranged from 3 to 84 months, with a median of 6 months, with 91.7% being less than 12 months old and 45.8% less than 6 months old.

On physical examination, solitary (n = 1) or multiple (n = 23) erythematous non-pruritic papules were observed in non-haired skin areas (Table 1).

The onset of clinical lesions was mainly in autumn (70.8%), followed by winter (16.7%), spring (8.3%), and summer (4.2%) (Table 2).

No other abnormalities were found on physical examination, except a mild lymphadenomegaly of regional lymph nodes in two dogs.

Most dogs showed no laboratory abnormalities except for one dog with a mild increase in TP and three dogs with a mild decrease in TP.

Clinical characteristics and laboratory tests according to different treatments at the time of diagnosis are displayed in Table 3.

### 3.2. Safety and Clinical Efficacy of Treatments

All treatments studied were considered safe. No side effects (local or systemic) were observed during the treatment period in any formulations studied.

Regarding the PHMB formulations, there were no statistically significant differences between the PHMB alone group and the PHMB with TLR4a at day 15 (chi-square: χ^2^ = 0.9; df = 2, *p* = 0.64) and day 30 (chi-square: χ^2^ = 3.6; df = 2, *p* = 0.17). On the other hand, although differences between local administration of PHMB alone or in combination with TLR4a when compared to the placebo administration were not statistically significant, and thus it is unclear if this would reflect on a large canine population, the results suggested a higher tendency towards resolution (both partial and total) at day 15 (chi-square: χ^2^ = 5.78; df = 2, *p* = 0.06) and day 30 post-treatment (chi-square: χ^2^ = 4.; df = 2, *p* = 0.12). Conversely, dogs treated with local administration of meglumine antimoniate had a statistically significant fastest resolution compared with placebo treatment at 15 (chi-square: χ^2^ = 12.58; df = 2, *p* = 0.002) and 30 days post-treatment (chi-square: χ^2^ = 9.47; df = 2, *p* = 0.009). Although no statistically significant differences were observed between the administration of meglumine antimoniate and the administration of PHMB alone or in combination with TLR4a at 15 days post-treatment (chi-square: χ^2^ = 2.57; df = 2, *p* = 0.27), there was a higher tendency towards resolution at 30 days post-treatment (chi-square: χ^2^ = 4.74; df = 2, *p* = 0.09) when meglumine antimoniate was administered (Table 4).

Regarding the dogs included in the control group (n = 9), some showed a total resolution of the papules at days 30 (n = 1/9), 90 (n = 2/9), and 180 (n = 1/9), two were excluded from the study at day 30 because of clinical worsening, and they were treated with meglumine antimoniate subcutaneously (2/9). The other three dogs (3/9) showed a partial resolution at day 60, but they were lost to follow-up (Figure 3).

### 3.3. Clinical Worsening (Withdrawal) and Lost to Follow-Up

Some dogs were lost to follow-up or withdrawn from the study for several reasons (Figure 3). In four (two from the control group and two from the PHMB + TLR4a group), other clinical signs such as loss of body weight, generalized lymphadenomegaly, and decreased appetite were observed during the follow-up, and *L. infantum*-specific antibody levels increased. They were treated with meglumine antimoniate subcutaneously and withdrawn from the study from that point.

Samples from several dogs could not be collected during the lockdown caused by SARS-CoV-2, but they were collected again in further follow-ups (Figure 3).

In one dog treated with PHMB, initially, clinical resolution of papules was observed, although new papules appeared during the treatment. This was considered a relapse, and a local rescue therapy (30% meglumine antimoniate + 70% pluronic F-127) was instituted. In this case, papules were all resolved on day 15 after the rescue therapy was started.

### 3.4. Leishmania Infantum-Specific Antibody Levels at the Time of Diagnosis and Follow-Up

The results of *L. infantum*-specific antibody levels at the time of diagnosis and during treatment follow-up are displayed in Table 5.

At the time of diagnosis, all dogs presented negative or low antibody levels except one dog (n = 24, median: 21.4, ranging from 2.8 to 227.1 EU). At day 0, six dogs were positive against *L. infantum* antigen, five of which were low positive, and one was medium positive. The rest of the dogs (n = 18) were seronegative. At day 15 (n = 23, median: 17.8, ranging from 3.5 to 167.4 EU), seven dogs were low positive, one dog was medium positive, and the rest were seronegative. At day 30 (n = 20, median: 13.8, ranging from 2.9 to 121.6 EU), six dogs were low positive, and the rest were seronegative. At day 60 (n = 14, median: 13.3, ranging from 3.8 to 115.5 EU), four dogs were low positive, and the rest were seronegative. At day 90 (n = 15, median: 12.4, ranging from 2.8 to 161.6 EU), only one dog was medium positive, and the rest were seronegative. At day 180 (n = 13, median: 3, ranging from 2.8 to 26 EU), all dogs were seronegative. At day 365 (n = 13, median: 16.3, ranging from 2.4 to 213.7 EU), one dog was medium positive, and the rest were seronegative; this dog had not been tested at day 180 due to lack of compliance of the owner.

No significant differences were observed when comparing *L. infantum* antibody levels at day 0 with the follow-up at any time (*p* = 0.98). Regarding the follow-up, only in 10 dogs (41.7%) was it possible to complete it as planned (days 0, 15, 30, 60, 90, 180, and 365). At days 180 and 365, thirteen dogs out of a total of 24 (54.2%) were tested.

Twelve dogs remained seronegative, while only one dog remained seropositive during all the follow-ups. Interestingly, four dogs experimented with a seroreversion since the antibody levels changed from low positive to negative. In contrast, no dogs showed seroconversion during the follow-up period. One dog treated with PHMB + TLR4a changed from low positive to medium positive.

### 3.5. IFN-γ Concentration at the Time of Diagnosis and During Treatment Follow-Up

The results of IFN-γ concentration at the time of diagnosis and during treatment follow-up are displayed in Table 5.

**Figure 4:**
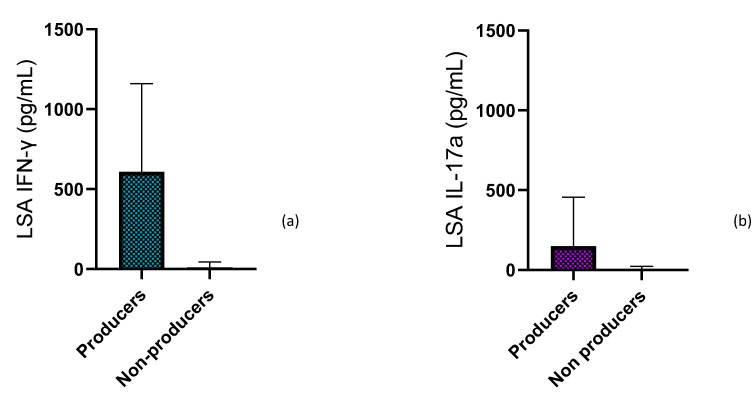
Median concentration with 95% CI (**a**) IFN-γ and (**b**) IL-17a concentration after LSA stimulation in cytokine producers and non-producers dogs at the time of diagnosis and prior to administration of local treatment.

At day 0, 12 of 24 dogs were considered IFN-γ producers. Supernatants from LSA-stimulated whole blood of IFN-γ producer dogs presented significantly higher concentrations of IFN-γ (median: 607.8, ranging from 171.9 to 3997.5 pg/mL) when compared with IFN-γ non-producers (median: 10.6, ranging from 0 to 84 pg/mL) (*p* < 0.0001) (Figure 4a). However, the concentration of IFN-γ of ConA stimulated blood from IFN-γ non-producers (median: 1287, ranging from 153.8 to 4254.2 pg/mL) did not show statistically significant differences (*p* = 0.442) with the IFN-γ producers’ group (median: 1287.5, ranging from 459.3 to 5959.9 pg/mL).

At day 30, 9 of the 20 dogs analyzed were classified as IFN-γ producers. Supernatants from LSA-stimulated whole blood of IFN-γ producer dogs showed higher concentrations of IFN-γ (median: 668.9, ranging from 233.9 to 3614 pg/mL) when compared with the IFN-γ non-producers’ group (median: 23.6, ranging from 0 to 62.7 pg/mL) (*p* = 0.0017). Regarding the concentration of IFN-γ of ConA stimulated blood from IFN-γ producers (median: 2503.7, ranging from 357.3 to 5073.2 pg/mL), there were no significant differences (*p* = 0.14) with the IFN-γ non-producers (median: 1295.6, ranging from 293.8 to 3576 pg/mL).

Concerning the rest of the follow-up, 9 dogs out of a total of 12 were considered IFN-γ producers at day 90, 5 out of 11 dogs were producers at day 180, while 5 dogs out of 12 remained IFN-γ producers at day 365.

Additionally, no statistically significant differences were observed when comparing IFN-γ concentrations in supernatants of blood stimulated with LSA from all dogs at day 0 and day 30 (*p* = 0.35) or any other time point during follow-up nor when comparing IFN-γ concentration after ConA stimulation at day 0 and day 30 (*p* = 0.81).

### 3.6. IL-17a Concentration at Time of Diagnosis and During Treatment Follow-Up

The results of IL-17a concentration at the time of diagnosis and during treatment follow-up are displayed in Table 5.

At diagnosis, 10 of 24 dogs were classified as IL-17a producers. Supernatants from LSA-stimulated whole blood of IL-17a producer dogs presented significantly higher concentrations of IL-17a (median: 149.5, ranging from 69.4 to 1933.3 pg/mL) when comparing with IL-17a non-producers (median: 0, ranging from 0 to 32.1 pg/mL) (*p* < 0.0001) (Figure 4b). However, concentrations of IL-17a of ConA stimulated blood from IL-17a non-producers (median: 6466.3, ranging from 721.3 to 23,050.2 pg/mL) did not show statistically significant differences (*p* = 0.34) with the IL-17a producers’ group (median: 9228.7 ranging from 3363.5 to 18,143.3 pg/mL).

On day 30, 11 dogs were considered IL-17a producers out of 20 dogs. IL-17a concentration after LSA stimulation of IL-17a producer dogs demonstrated higher concentrations of IL-17a (median: 155.7 ranging from 74 to 630.3 pg/mL) when comparing with the IL-17a non-producers’ group (median: 2.8 ranging from 0 to 40 pg/mL) (*p* < 0.0001). IL-17a concentration after ConA stimulation was not statistically different (*p* = 0.56) between IL-17a producers (median: 14,088.6 ranging from 1082.1 to 41,390 pg/mL) and IL-17a non-producers (median: 12,425.3 ranging from 2637 to 31,150 pg/mL) dogs.

Furthermore, no statistically significant differences were observed when comparing IL-17a concentrations in supernatants of blood stimulated with LSA from all dogs at day 0 and day 30 (*p* = 0.08). However, significant differences were found when comparing IL-17a concentration after ConA stimulation at day 0 and day 30 (*p* = 0.01).

### 3.7. Correlation between Parameters Studied

Considering all dogs studied (n = 24), a correlation was not found between IFN-γ concentration after LSA stimulation and *L. infantum* specific antibody levels (Spearman r: 0.31, *p* = 0.15) neither between IL-17a after LSA stimulation and antibody levels (Spearman r: 0.25, *p* = 0.24). Moreover, there was no correlation between the age of the dogs and antibody levels (Spearman r: −0.28, *p* = 0.18).

No correlation was found between PCR Cq and *L. infantum* specific antibody levels (Spearman r: −0.07, *p* = 0.8) nor PCR Cq and IFN-γ concentration after LSA stimulation (Spearman r: 0.41, *p* = 0.09) nor between PCR Cq and IL-17a after LSA stimulation (Spearman r: 0.46, *p* = 0.06).

**Figure 5:**
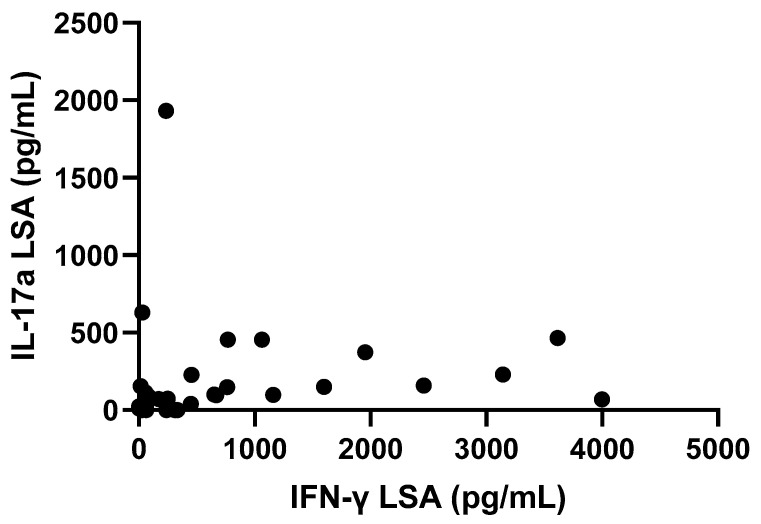
Correlation between IL-17a (pg/mL) and IFN-γ (pg/mL) after LSA stimulation in the total of dogs studied at the time of diagnosis and follow-up. Spearman r: 0.59, *p* < 0.0001.

IFN-γ concentration after LSA stimulation was significantly positively correlated with IL-17a concentration after LSA stimulation (Spearman r: 0.59, *p* < 0.0001) (Figure 5).

Similarly, there was a significant correlation between IFN-γ concentration after Con A stimulation and IL-17a concentration after Con A stimulation (Spearman r: 0.54, *p* = 0.0002).

### 3.8. Nitroblue Tetrazolium Reduction Test

There were no statistically significant differences when comparing the NBT reduction rate at day 0 (mean ± SD: 18 ± 6%) with the NBT reduction rate at day 30 (mean ± SD: 19 ± 5%) (*p* = 0.53).

### 3.9. Blood PCR

The *Leishmania* real-time PCR results at the time of diagnosis and during the follow-up period are depicted in Table 5.

At the time of diagnosis, 13 out of a total of 24 (54.2%) dogs were PCR positive.

At day 30, only 4 dogs were PCR positive from a total of 20 (20%) dogs.

Interestingly, 2 dogs remained positive from day 0 to day 30. The other 2 negative dogs at day 0 were PCR positive at day 30. In contrast, 8 dogs that were PCR positive at day 0 were negative at day 30. Unfortunately, 4 dogs were lost to follow-up on day 30.

Regarding the last PCR performed from each dog, one was at day 60 and was positive, while it was negative at days 0 and 30.

On day 180, 3 dogs were tested, and all were PCR positive.

Finally, at day 365, 12 out of 13 dogs were PCR positive (92%). Two dogs remained always positive from day 0, only one remained negative from day 0, and the others changed depending on the time.

Regarding the proportion of positive dogs, significant differences were found when comparing day 0 to day 30 (*p* = 0.03), day 0 to day 365 (*p* = 0.02), and day 30 to day 365 (*p* ≤ 0.0001). Statistically significant differences were observed when comparing PCR Cq results at day 0 (mean ± SD: 34.7 ± 2.1) and day 30 (mean ± SD: 37.1 ± 0.5) (*p* = 0.04), and when comparing PCR Cq results at day 30 and day 365 (mean ± SD: 34.7 ± 1.2) (*p* = 0.0023). However, no differences were observed when PCR Cq results at day 0 and day 365 were compared (*p* = 0.93).

## 4. Discussion

To the author’s best knowledge, there are few studies evaluating the effectiveness of local treatment in canine cutaneous leishmaniosis (CCL), but no studies so far have evaluated local treatment of papular dermatitis due to *L. infantum* infection [40,41,42]. In the present randomized controlled study, 70% of dogs treated with meglumine antimoniate and 40% of dogs treated with PHMB alone showed a complete response after 30 days of treatment, demonstrating a much faster response than the median of 98 ± 5 days observed in a previous observational study. However, it is difficult to compare these results as we only included dogs with stage I-mild disease-papular dermatitis, and in the previous study, all dogs had ulcerative lesions in which self-healing was not expected [1,40]. Moreover, no adverse effects were observed throughout the course of the study in any of the dogs. Therefore, we might conclude that all the studied substances were safe.

Although meglumine antimoniate is widely described as a systemic treatment for *L. infantum* infection both in humans and dogs [36], to the author’s best knowledge, there is limited information regarding the efficacy of its topical use in dogs with either cutaneous leishmaniosis or papular dermatitis. Interestingly, a recent study demonstrated the efficacy of an intralesional meglumine antimoniate compared with a placebo for the treatment of CCL due to *L. braziliensis* [41]. Dogs with localized cutaneous ulcers were enrolled in this study and showed a higher and faster healing rate when they were treated with intralesional meglumine antimoniate [41]. In human medicine, meglumine antimoniate is commonly used as a local treatment through intralesional administration and is considered appropriate for the treatment of simple cutaneous lesions due to *L. panamensis* and *L. mexicana* [43]. Local application of meglumine antimoniate has also been tested in murine leishmaniosis models [44,45,46,47]. The main obstacle to the local administration of meglumine antimoniate is that it is considered a water-soluble molecule, and thus, it has limited interaction with lipophilic skin structures [29].

In murine models, different drug delivery systems have been evaluated. Moosavian Kalat et al. (2014) tested a liposome containing 22.5% meglumine antimoniate as a local treatment on infected BALB/c mice and observed a reduction in lesion size and a lower spleen parasite burden in treated mice compared to the control group [44]. The same group, in a further study (2019), tested a similar mixture by adding stearylamine, which improved liposome performance with good clinical improvement [45]. In another study published in 2013, meglumine antimoniate administered using a liposomal formulation showed a reduction in lesion size and amastigote counts, although it had no significant therapeutic difference compared to the control group [46]. Finally, Horoiwaone et al. (2020) tested the local application of meglumine antimoniate contained in maltodextrin polymeric colloidal nanocarriers in a murine leishmaniasis model, which showed similar efficacy to the intraperitoneal injection regarding parasite titer reduction and superior healing activity in terms of collagen area deposition [47]. Other delivery systems have also been tested for systemic administration of meglumine antimoniate, which could be considered for topical administration in future studies, including polymeric nanoformulations such as aqueous-core poly-L-lactide nanocapsules, which have shown a great antileishmanial activity on mice infected with *L. infantum* [48]. In addition, this new formulation promotes meglumine antimoniate uptake within the macrophages, increasing its efficacy and decreasing its cellular-negative outcome [48].

In the present study, the administration of meglumine antimoniate with pluronic F-127 as a delivery system was tested, which is a non-ionic detergent used to facilitate the solubilization of water-insoluble materials in physiological media. A significant improvement was observed in our patients when compared to the control group, and no adverse effects were detected during the length of administration nor the posterior follow-ups, which could mean that pluronic F-127 is a safe and adequate delivery system and that locally administered meglumine antimoniate could be considered as a viable treatment option in dogs with localized papular dermatitis due to *L. infantum*. This new local formulation of meglumine antimoniate has shown to be safe for its use in humans, and it has been previously tested in vitro in three cell lines, ex vivo using human skin samples, and in vivo testing on human volunteers [35].

Regarding PHMB, to the author’s best knowledge, there are no previous in vivo studies reporting its use as a topical formulation for the treatment of papules due to *L. infantum* infection, neither in dogs nor in humans. On the other hand, it has been described in previous publications as a topical antimicrobial for locally infected wounds in dogs [49,50], and there is a study about the safety of its use in combination with other drugs as an ear flush in dogs [51]. In humans, PHMB has been used as a first-line treatment for infected wounds [52,53] and as a treatment for *Acanthamoeba* keratitis [54].

As mentioned previously, in the present study, dogs treated with topical PHMB showed a higher tendency toward resolution compared with the control group, although the results were not statistically significant. PHMB has been shown to have antileishmanial properties, such as disruption of the parasite´s membrane integrity and chromosomal damage, in previous in vitro studies [22,34]. We also evaluated its combination with TLR4a, as according to a previous study, TLR4 activation leads to the parasite’s death by inducing a protective immune response against *Leishmania* and regulating iNOS [22]. According to our results, no statistically significant differences were observed between the PHMB alone group and the PHMB combined with TLR4a. This lack of difference could be due to a lack of effectiveness regarding the TLR4a stimulation, insufficient dosage, or due to complications such as lack of absorption of the TLR4a. However, a small number of dogs were included in these groups, and thus a significant difference could have been observed if more dogs were included.

To the author’s best knowledge, there are only two published studies in which a follow-up over dogs with papular dermatitis was performed [4,55]. In one of them, 15 out of 17 dogs were treated with subcutaneous meglumine antimoniate for 25–30 days, showing complete resolution of lesions by day 25 in all dogs [4]. The remaining dog received no treatment and was only revisited four months later, showing only partial remission [4]. In the other study, 3 out of 8 dogs were treated twice a day with meglumine antimoniate subcutaneously (50 mg/kg SC BID) and allopurinol orally (10 mg/kg PO BID), showing total resolution by 10 days of treatment [55]. The other four dogs received no treatment, showing improvement only after 3 to 5 months [55]. Similarly, in the present study, all dogs treated with topical meglumine antimoniate showed improvement one month after starting treatment, although only 70% had a complete resolution of lesions. However, taking into account the mean of administration (much less painful and stressful for the dog and the owner) and the lower risk of adverse effects, we can consider topical treatment as an advantageous alternative.

As mentioned previously, self-healing of the skin lesions may occur between three and six months in previous studies [4,55]. This fact is similar to the results of the present study, in which some dogs included in the control group showed a total resolution of the papules at days 90 (22%) and 180 (11%) or partial resolution at day 60 (11%), although one showed total resolution by day 30 (11%).

The immune response was also evaluated in this study. IFN-γ is one of the cytokines expressed by a Th1 response (considered an effective immune response). In the present study, 50% (12/24) of dogs at day 0 and 45% (9/20) at day 30 were considered IFN- γ producers. These findings are similar to previous studies using IFN-γ release whole blood assay in dogs with stage I and papular dermatitis. In one study [5], dogs in stage I (25.7%) and IIa (48.5%) showed a higher production of IFN-γ than other dogs classified as stage IIb (8.5%), III (14.2%), and IV (2.8%). In another study, 15 of 19 dogs in stage I (79%) were IFN-γ producers, whereas only 6 out of 15 dogs (40%) were in stage II-III [6]. Interestingly, in this study, the dogs that were followed up at different time points presented a production of parasite-specific IFN-γ that ranged from 41 to 75% of the dogs studied.

The results of IFN-γ concentration showed a positive correlation with IL-17a concentration after LSA and Con A stimulation. This correlation was expected regarding previous studies where it was demonstrated that IL-17a played a synergic role with IFN-γ by blocking parasite growth after checking IFN-γ and IL-17a levels in infected mice [10]. In another study, mRNA expression for iNOS and IFN- γ was positively correlated with IL-17a gene transcription in dogs [11]. However, another study concluded that SNPs located in analogous regions of canine IL-17a gene promoter did not show an association with *Leishmania* spp. resistance [56]. Further studies on the role played by IL-17a in *L. infantum* infection are required to determine if it might be used as a prognostic marker.

In addition, it is important to highlight that, similarly to previous studies, the majority of these dogs were seronegative or presented very low positive antibody levels throughout the study period, and four dogs even showed seroreversion [6]. Therefore, even if dogs were only treated with local treatment or no treatment, most of them did not have relapses or worsening of the infection. Regarding this, only one dog treated with PHMB alone showed relapse of papular dermatitis, another dog treated with PHMB + TLR4a demonstrated an increase in serology levels through treatment, and four dogs were withdrawn from the study due to worsening of clinical signs, two associated to the control group (2/4) and two to PHMB + TLR4a group (2/4). Conversely, no dogs treated with local meglumine antimoniate showed worsening or relapse of clinical signs throughout the study period, nor an increase in antibody levels, and the dog that had relapsed while PHMB was being administrated, did show complete resolution after changing to local meglumine antimoniate, demonstrating, in these cases, a good efficacy not only in the treatment of clinical signs but also to avoid relapses or worsening of the disease.

According to previous papers, in this study, there was no association between sex or age and NBT reduction rate [24,25]. In a previous study, the NBT reduction rate of 40 healthy dogs (4.57 ± 1.72%), 20 dogs in Stage-I (34 ± 10.05%), and 20 dogs in Stage-IV (3.7 ± 2.03%) was compared, showing that NBT reduction rate was significantly higher in dogs in Stage-I [24]. In another study, the NBT reduction rate was compared between ten healthy dogs treated with 0.5 mg/kg oral (PO) of domperidone once a day (SID) for 30 days and ten non-treated healthy dogs [25]. In this previous study, the results from the non-treated group showed similar means to the first study (5.9 ± 1.6%) [25]. According to these previous papers, our results of NBT reduction rate at day 0 (mean ± SD: 18 ± 6%) and day 30 (mean ± SD: 19 ± 5%) were closer to the Stage-I results [24,25].

Concerning real-time PCR, the results of the present study are in agreement with previous studies where dogs with mild leishmaniosis showed a lower proportion of positive cases than dogs in more advanced stages [5,6,57]. It is well known that sick dogs with high antibody levels also show high parasitemia levels [57]. It is worth noticing that a difference was observed between the proportion of PCR-positive dogs at day 0 (54.2%) and day 30 (20%) (*p* = 0.03), and when comparing dogs at day 30 and day 365 (92.3%) (*p* = 0.0001), showing a significantly lower result at day 30 and a significantly higher result at day 365. This could be interpreted as a signal of infection recrudescence. However, it was not associated with clinical or analytical worsening, and the parasitemia (PCR Cq) was the same at day 365 (34.7 ± 1.2) and at day 0 (34.7 ± 2.1), indicating a possible clinical cure with infection persistence or progression. Nevertheless, it should be taken into account that in this study, dogs were changing between PCR positive or negative depending on the day of the follow-up. Intermittent or transitory parasitemia has been commonly observed in previous studies in dogs with *Leishmania* infection and is the most likely explanation for the variability of the results throughout the follow-up [58]. Moreover, the number of dogs that were available for PCR testing on day 365 was much lower than initially, and this could affect the results. It should also be pointed out that PCR performed on DNA extracted from whole blood samples is a less sensitive technique than PCR performed on bone marrow, lymph node, spleen, or skin, which could be regarded as a limitation of this study [59,60]. Invasive sampling for PCR was considered not ethical in this scenario.

Regarding the onset of clinical signs, 87.5% of the papules appeared in autumn and winter, as previously described [4,61]. This period belongs to the end of the sandfly season, which could mean a delay between *Leishmania* inoculation and the development of papular dermatitis. This might be explained by a period of parasite amplification, as it was described in mice [62] and dogs [63]. The majority of dogs in this study were young, 91.7% were below 12 months of age, and 45.8% were below 6 months of age, as was observed in previous studies [4,61]. This could mean that papular dermatitis might be more common at the time of the first contact of the parasite with the host’s immune system. In the same way, the distribution of papular lesions was similar to these previous studies, 46.9% were located on the inner surface of the pinna, and 18.8% were on the abdomen [4,61].

## 5. Conclusions

In conclusion, the results of this study showed that dogs with papular dermatitis treated with local meglumine antimoniate healed faster than dogs treated with a placebo. Furthermore, no dog treated with topical meglumine antimoniate showed worsening clinical signs or relapse during the one-year follow-up. On the other hand, PHMB (alone or in combination with TLR4a) showed a higher tendency towards resolution when compared to placebo, although results were considered non-significant. Moreover, no adverse effects were observed in any of the drugs throughout the study in any of the dogs. Therefore, we might conclude that local meglumine antimoniate is a safe and effective alternative for the treatment of papular dermatitis in dogs with *L. infantum* infection. The present study also showed that most dogs presented a protective immune response at the time of diagnosis and during a year follow-up period without clinical failure. 

## Figures and Tables

**Table 1:** Distribution of papules.

Site	Number of Lesions	%
Inner surface of the pinna	15	46.9
Abdomen	6	18.8
Eyelid	3	9.4
Lips	3	9.4
Foreskin	2	6.3
Elbow	1	3.1
Bridge of the nose	1	3.1
Nose	1	3.1

**Table 2:** The onset of clinical lesions was analysed by season, sex, and median age.

	Total Number of Dogs	%	Median Age (Minimum-Maximum Months)
**Autumn**	17	70.8	6 (3–84)
Female	5	20.8	7 (3–84)
Male	12	50	6 (4–6)
**Winter**	4	16.7	6 (6–10)
Female	3	12.5	6 (6–10)
Male	1	4.2	5
**Spring**	2	8.3	3.5 (3–4)
Male	2	8.3	3.5 (3–4)
**Summer**	1	4.2	5
Female	1	4.2	5
**Total**	24	100	6

**Table 3:** Clinical characteristics and laboratory tests at the time of diagnosis before starting local treatment.

**Qualitative Characteristics**		**PHMB + PHMB with TLR4a (n = 9)**	**Meglumine** **Antimoniate** (n = 6/10) *	**Control Group (n = 9)**	***p*** **-Value** **(Fisher’s** **Exact Test)**
Breed	Crossbred	7 (78%)	3 (50%)	3 (33%)	0.162
	Purebred	2 (22%)	3 (50%)	6 (67%)	
Sex	Male	7 (78%)	2 (33%)	5 (56%)	0.227
	Female	2 (22%)	4 (67%)	4 (44%)	
ELISA at day 0	Low positive	3 (33%)	0 (0%)	3 (33%)	0.264
	Negative	6 (67%)	6 (100%)	6 (67%)	
*Leishmania* real-time PCR	Positive	5 (56%)	3 (50%)	5 (56%)	0.972
	Negative	4 (44%)	3 (50%)	4 (44%)	
IFN-γ	Producers	7 (78%)	3 (50%)	2 (22%)	0.1
	Non-prod.	2 (22%)	3 (50%)	7 (78%)	
IL-17a	Producers	8 (89%)	1 (17%)	1 (11%)	0.7
	Non-prod.	1 (11%)	5 (83%)	8 (89%)	
**Quantitative Characteristics**		**Median** **(min-max)**	**Median** **(min-max)**	**Median** **(min-max)**	***p*** **-Value** **(Kruskal-Wallis Test)**
Age (months)		6 (3–8)	5.5 (4–84)	6 (3–36)	0.875
IFN-γ (pg/mL)		768.6 (11.6–3997.5)	140.4 (0–762.4)	23.6 (0–309)	0.011 **
IL-17a (pg/mL)		151.1 (0–1933.3)	11.9 (0.148)	0 (0–71.5)	0.005 **
ELISA Units		26 (7.1–227.1)	5.7 (2.8–73.5)	22.3 (4.8–100.9)	0.11
**Quantitative Characteristics**		**Mean ± SD**	**Mean ± SD**	**Mean ± SD**	***p*** **-Value** **(Ordinary One-Way ANOVA)**
Cq PCR		35.3 ± 1.9	35 ± 1.4	33.9 ± 2.7	0.58

PHMB = polyhexamethylene biguanide, TLR4a = Toll-like receptor 4 agonist, IFN-γ = Interferon-gamma, IL-17a = Interleukin-17a, Cq = Quantification cycle, Non-prod= non-producers. * Complete data were only obtained from 6 out of 10 dogs in this group. ** Significant difference was observed between PHMB + PHMB with TLR4a and control groups but not between PHMB + PHMB with TLR4 and meglumine antimoniate groups and between meglumine antimoniate and control groups.

**Table 4:** Clinical resolution of papules according to the different treatments (PHMB alone, PHMB in combination with TLR4a, and meglumine antimoniate).

Local Treatment (Number of Dogs)	Clinical Resolution (Number of Dogs/Total, %)					
	15 Days Post-Treatment			30 Days Post-Treatment		
	Without Resolution	Partial	Total	Without Resolution	Partial	Total
**PHMB alone (n = 5)**	1/5, 20%	3/5, 60%	1/5, 20%	1/5, 20%	2/5, 40%	2/5, 40%
**PHMB+ TLR4a (n = 4)**	1/4, 25%	3/4, 75%	0/4, 0%	0/4, 0%	4/4, 100%	0/4, 0%
**Meglumine antimoniate (n = 10)**	0/10, 0%	8/10, 80%	2/10, 20%	0/10, 0%	3/10, 30%	7/10, 70%
**Control group (n = 9): diluent (n = 5) + TLR4a (n = 4)**	7/9, 78%	2/9, 22%	0/9, 0%	5/9, 56%	3/9, 33%	1/9, 11%
**Total (n = 28)**	9	16	3	6	12	10

PHMB = polyhexamethylene biguanide, TLR4a = Toll-like receptor 4 agonist.

**Table 5:** Serological, molecular, IFN-γ and IL-17a concentrations and NBT results of dogs at diagnosis and follow-up.

	Number of Seropositive Dogs (%)	Median ELISA Units (min-max)	Number of LSA IFN-γ Producers (%)	Median LSA IFN-γ (pg/mL) (min-max)	Number of LSA IL-17a Producers (%)	Median LSA IL-17a (pg/mL) (min-max)	PCR Positives (%)	Mean Cq ± SD	Mean NBT Rate ± SD (%)
**Day 0**	6/24 (25%)	21.4 (2.8–227.1)	12/24 (50%)	127.9 (0–3998)	10/24 (42%)	23.2 (0–1933)	13 (54.2)	34.7 ± 2.1	18 ± 6%
**Day 15**	8/23 (34.8%)	17.8 (3.5–167.4)	-	-	-	-			-
**Day 30**	6/20 (30%)	13.8 (2.9–121.6)	9/20 (45%)	61.7 (0–3614)	11/20 (55%)	85.2 (0–630.3)	4 (20)	37.1 ± 0.5	19± 5%
**Day 60**	4/14 (28.6%)	13.3 (3.8–115.5)	-	-	-	-			-
**Day 90**	1/15 (6.7%)	12.4 (2.8–161.6)	9/12 (75%)	444.1 (4.6–6002.9)					
**Day 180**	0/13 (0%)	9.58 (3–26)	5/11 (45.5%)	41.6 (0–2660)					
**Day 365**	1/13 (8%)	16.3 (2.4–213.7)	5/12 (41.7%)	1250.2 (0–6947.7)			12 (92.3)	34.7 ± 1.2	

Cq = Quantification cycle, IFN-γ = Interferon-gamma, IL-17a = Interleukin-17a, LSA = soluble *L. infantum* antigen, NBT = Nitroblue tetrazolium, min = minimum, max = maximum.

## Data Availability

Data supporting reported results can be obtained by emailing the corresponding author.

## Supplementary Materials

The following supporting information can be downloaded at: https://www.mdpi.com/article/10.3390/pathogens12060821/s1, Table S1: Incident sheet for owner (treatment application, adverse effects, or others).
