# Supplementary material for: Effect of Local Administration of Meglumine Antimoniate and Polyhexamethylene Biguanide Alone or in Combination with a Toll-like Receptor 4 Agonist for the Treatment of Papular Dermatitis due to Leishmania infantum in Dogs
Source: Pathogens. 2023 Jun 10;12(6):821. doi: 10.3390/pathogens12060821 (PMC10304710; doi:10.3390/pathogens12060821)
Supplement: Supplementary file 1 [file pathogens-12-00821-s001.zip › pathogens-2340783-Table S1.pdf]

INCIDENT SHEET FOR OWNER (treatment application, adverse effects, or others)

Dog ID

Detection date (days after treatment)												
Erythema / crusts (indicate from 0 to 4)												
Edema (indicate from 0 to 4)												
Itching (indicate from 0 to 4)												
Pain (indicate ‘yes’ or ‘no’)												
Other skin lesions (mark with an X the day of visit)*												
Other clinical signs (mark with an X the day of visit)*												
Treatment application (indicate ‘yes’ or ‘no’)**												

* Indicate on the incident sheet of the corresponding visit (1, 2, 3, or 4)

** In the case that the treatment could NOT be applied, describe it in this incident sheet.

Day of visit: The treatment has not been applied for the following reason:

ADVERSE EFFECTS BY VETERINARIAN

Day of visit ..../……../…….. **Dog ID** ☐

ERYTHEMA / CRUSTS **0 1 2 3 4**

EDEMA **0 1 2 3 4**

ITCHING **NO SI**

PAIN **NO SI**

OTHER SKIN LESIONS OR OTHER CLINICAL SIGNS (SPECIFY):

**Erythema and crusts**	**Formation of edemas**	
Very slight erythema	very slight edema (barely noticeable)	1
Well-defined erythema	slight edema (edges of the area well defined by elevation)	2
Moderate to severe erythema	Moderate edema (elevation of approximately 1 mm)	3
Severe or crusts formation	Severe edema (elevation of more than 1 mm, extending beyond the exposure site)	4

PHOTO FOR ADVERSE EFFECT ASSESSMENT ☐

WITHDRAWAL FROM THE STUDY AND REASON

Day of visit ..../……../……   **Dog ID**

**WITHDRAWAL FROM THE STUDY**	**YES**	**NO**
**REASON**		

VETERINARIAN

Signature: ______________ **Date:** ____/____/____
